# Exploring the xylose paradox in *Saccharomyces cerevisiae* through in vivo sugar signalomics of targeted deletants

**DOI:** 10.1186/s12934-019-1141-x

**Published:** 2019-05-23

**Authors:** Karen O. Osiro, Celina Borgström, Daniel P. Brink, Birta Líf Fjölnisdóttir, Marie F. Gorwa-Grauslund

**Affiliations:** Applied Microbiology, Department of Chemistry, Lund University, Lund, Sweden

**Keywords:** *Saccharomyces cerevisiae*, Sugar sensing/signalling, Xylose, GFP biosensor, cAMP/PKA, Snf3p/Rgt2p, SNF1/Mig1p, ∆*ira2*, ∆*isu1*, ∆*hog1*

## Abstract

**Background:**

There have been many successful strategies to implement xylose metabolism in *Saccharomyces cerevisiae*, but no effort has so far enabled xylose utilization at rates comparable to that of glucose (the preferred sugar of this yeast). Many studies have pointed towards the engineered yeast not sensing that xylose is a fermentable carbon source despite growing and fermenting on it, which is paradoxical. We have previously used fluorescent biosensor strains to in vivo monitor the sugar signalome in yeast engineered with xylose reductase and xylitol dehydrogenase (XR/XDH) and have established that *S. cerevisiae* senses high concentrations of xylose with the same signal as low concentration of glucose, which may explain the poor utilization.

**Results:**

In the present study, we evaluated the effects of three deletions (*ira2*∆, *isu1*∆ and *hog1*∆) that have recently been shown to display epistatic effects on a xylose isomerase (XI) strain. Through aerobic and anaerobic characterization, we showed that the proposed effects in XI strains were for the most part also applicable in the XR/XDH background. The *ira2*∆*isu1*∆ double deletion led to strains with the highest specific xylose consumption- and ethanol production rates but also the lowest biomass titre. The signalling response revealed that *ira2*∆*isu1*∆ changed the *low glucose*-signal in the background strain to a simultaneous signalling of high and low glucose, suggesting that engineering of the signalome can improve xylose utilization.

**Conclusions:**

The study was able to correlate the previously proposed beneficial effects of *ira2*∆, *isu1*∆ and *hog1*∆ on *S. cerevisiae* xylose uptake, with a change in the sugar signalome. This is in line with our previous hypothesis that the key to resolve the xylose paradox lies in the sugar sensing and signalling networks. These results indicate that the future engineering targets for improved xylose utilization should probably be sought not in the metabolic networks, but in the signalling ones.

**Electronic supplementary material:**

The online version of this article (10.1186/s12934-019-1141-x) contains supplementary material, which is available to authorized users.

## Introduction

Microbial fermentation and bioconversion can be used for sustainable production of bulk and fine chemicals from renewable feedstocks. Of particular interest is fermentation of lignocellulose, a non-edible plant matter which is found in e.g. forestry and agricultural residues and municipal paper waste and that, unlike fermentation of crops such as corn and sugarcane, does not compete for arable land [1]. An industrially feasible lignocellulose biorefinery will require a microbe that not only can withstand the harsh conditions in the lignocellulosic hydrolysate (e.g. low pH, osmotic stress and inhibitory compounds) but also is able to process all the sugars in the feedstock, i.e. both hexose (C_6_) and pentose (C_5_) sugars [2, 3]. One of the most commonly used microorganisms in this context is Baker’s yeast *Saccharomyces cerevisiae*, which can be used to produce e.g. bioethanol from lignocellulosic hydrolysates since it naturally ferments glucose at high efficiency and has a basal inherent robustness to several of the stressors in the hydrolysate [4, 5]. However, wild type *S. cerevisiae* cannot utilize pentose sugars [6], and there is therefore a large interest to metabolically engineer this yeast to rapidly catabolize C_5_-sugars in general, and xylose—the second most abundant sugar in nature [7]—in particular.

There are currently two successfully implemented strategies for xylose utilization by *S. cerevisiae:* the oxido-reductive pathway [8, 9] and the isomerase pathway [10, 11]. The oxido-reductive strategy uses a xylose reductase (XR) and a xylitol dehydrogenase (XDH) to convert xylose to xylitol and xylitol to xylulose respectively [9]. The reactions are NAD(P)H-dependent, meaning that the cellular redox balance has to be considered when implementing this pathway. The isomerase strategy, on the other hand, relies on a xylose isomerase (XI) that converts xylose directly to xylulose without the requirement of any cofactors [10]. The XI is however easily inhibited by xylitol formation from endogenous reductases acting on the xylose, such as Gre3p [10]. Commonly, XR/XDH pathways are of fungal origin, whereas XI is bacterial [12], meaning that the former genes are more straightforward to express in *S. cerevisiae*. From xylulose, the endogenous xylulokinase (XK) will shunt the carbon into the non-oxidative part of the pentose phosphate pathway (PPP), where it will eventually reach the central carbon metabolism [9]. Xylose catabolism can be improved by modification of a number of endogenous genes in the PPP, such as overexpression of *XKS1* (encoding XK) [13, 14], *TAL1* and *TKL1* [15], and deletion of *GRE3* [16, 17] and *PHO13* [18, 19]. It can also be noted that the redox issue in the XR/XDH pathway can be improved by the expression of an XR with preference for NADH over NADPH [20], and that novel XIs that are less inhibited by xylitol have been discovered [21]. Deletion of the *GRE3* reductase is also beneficial for XIs, as *gre3*∆ results in less endogenous xylitol formation from xylose [22]. Xylose uptake is another issue in *S. cerevisiae*: there are no specialized xylose transporters and the uptake takes place through hexose transporters that have some affinity for xylose, but the simultaneous presence of glucose impairs xylose uptake [23]. Therefore some hexose and galactose transporters have been engineered for improved xylose specificity [24–27].

However, despite many successful metabolic engineering strategies, xylose utilization by recombinant *S. cerevisiae* is still lagging behind the performance on glucose. For example there are now strains with yields of ethanol from xylose [20, 28] close to the maximum theoretical yield of 0.51 g/g substrate [29], but the specific productivities on xylose of these strains are about 3–8 times lower than what is normally seen on glucose (typically around 2 g ethanol g cell dry weight^−1^ h^−1^ on glucose; [30]). Co-consumption of glucose and xylose is another issue, as xylose is typically only taken up after most of the glucose has been depleted [12, 31], which leads to inefficient fermentation times. Because of these phenotypes, it has been suggested that xylose triggers a non-fermentative response in the recombinant *S. cerevisiae* and evidenced by e.g. transcript and metabolite profiling and observed respiratory behaviour [32–37].

**Fig. 1:**
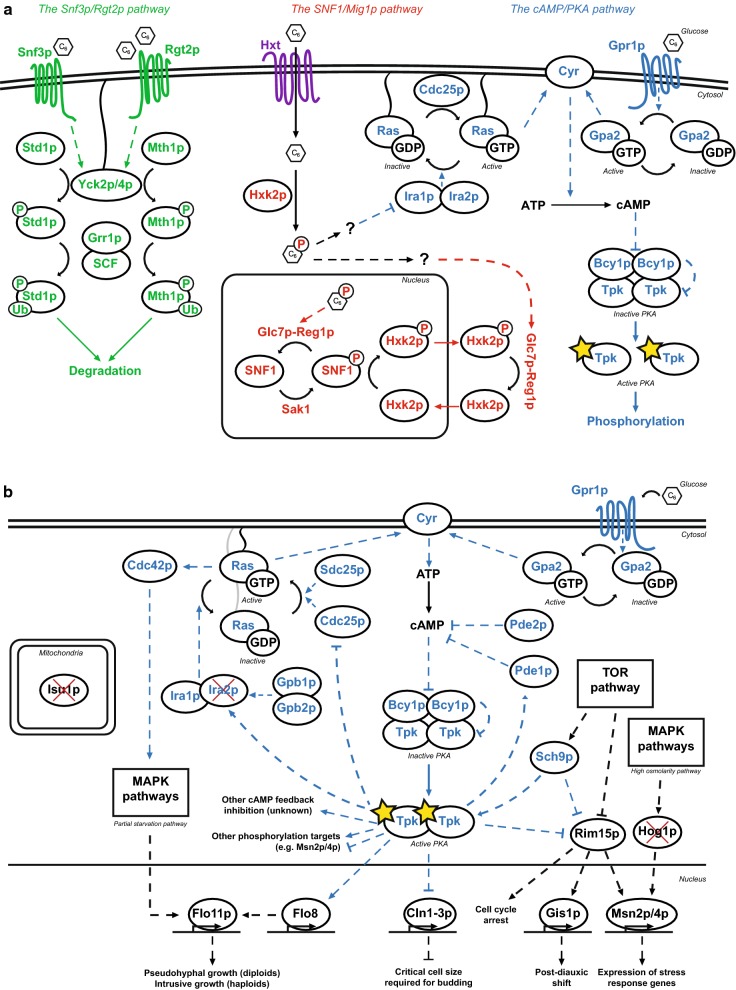
Schematic representation of the signalling pathways and deletions investigated in the current study. **a** The three main sugar signalling pathways in *S. cerevisiae*, adapted from [39]. The Snf3p/Rgt2p pathway (green) handles expression of hexose transporters in response to extracellular glucose. The SNF1/Mig1p pathway (red) handles expression of genes related to alternative (non-glucose) carbon sources in response to intracellular phosphorylated glucose. The cAMP/PKA pathway (blue) handles e.g. cellular growth, homeostasis and stress response. **b** Detailed schematic map of the cAMP/PKA pathway with the genes that were deleted in the current study marked with a red cross. Note that two of the three genes (*IRA2*, *HOG1*) are closely related to the pathway, whereas the third one (*ISU1*) is located in the mitochondria and seemingly unrelated to the cAMP/PKA pathway. **b** was adapted from [46, 47, 56, 63, 81–85]. Solid arrows represent reactions/transport and dashed arrows represent induction (arrowhead) or repression (hammerhead)

This xylose paradox—that xylose is fermented to ethanol despite the cellular signals suggesting otherwise—has led us to believe that the root of the poor productivity and co-consumption may be found in the sugar sensing and signalling pathways of *S. cerevisiae.* Previously, we constructed and validated a panel of in vivo fluorescent biosensors [38] that allows for single-cell real-time monitoring of the signals of the three main sugar sensing pathways in this yeast: the Snf3p/Rgt2p pathway, the SNF1/Mig1p pathway and the cAMP/protein kinase A (PKA) pathway (Fig. 1a). It was found that in *S. cerevisiae* strains that had not been engineered for xylose utilization, extracellular xylose did not trigger any signals, but a certain population heterogeneity on xylose indicated that there might be an endogenous sensing of intracellular xylose [38]. When the same biosensors were later applied to strains that had been engineered for xylose uptake with the XR/XDH pathway and a xylose transporter, high xylose concentrations triggered the same signal as low glucose concentrations did; this indicated that xylose resulted in the opposite signal to that of glucose and that it may trigger a starvation response rather than a fermentation response [39].

These previous findings led us to look in the literature for genetic modifications that may affect the sugar signalling pathways in favour of xylose, that we could assay further with our biosensor system. In a recent study by Sato and colleagues, adaptive evolution and reverse engineering was used to discover previously unknown epistatic interactions between different genes that, when deleted, improved xylose uptake and utilization: *HOG1*, *IRA2*, *ISU1* and *GRE3* [40]. Their results showed that combinations of these deletions led to improved growth, xylose consumption and specific ethanol productivity on strains engineered with the XI pathway during anaerobic conditions; they also observed that *isu1∆* and *hog1∆* enabled aerobic xylose respiration [40]. *IRA2* and *HOG1* are known to be connected to the sugar and stress signalling networks (cAMP/PKA pathway and MAP kinase (MAPK) cascades respectively) [41, 42] and are therefore along the lines of our hypothesis of the importance of the signalling networks for solving the xylose paradox (Fig. 1b). The *ISU1* gene, which encodes a mitochondrial Fe–S cluster scaffold protein [43, 44], had however not been previously connected to xylose metabolism, but was recently pinpointed by two independent studies [40, 45]. As for *GRE3*, it may primarily improve the XI pathway—by decreasing the xylitol concentration and thus XI inhibition [22].

In the current study, we investigated the effects of the deletions found by Sato et al. on the sugar signalling pathways by introducing them in our biosensor strains, with a focus on the epistatic interactions of *HOG1*, *IRA2*, and *ISU1*. In particular, we wanted to answer the following research questions: are the observed xylose fermentation improvements also achievable with XR/XDH? Did the improvement of xylose consumption come from a change in the sugar signalome (i.e. all the sugar signalling pathways in the cell)? We here report on the fermentation profiles and biosensor responses of the combinatorial deletions of these genes in three of our XR/XDH-equipped biosensor strain lines.

## Results

### Deletion of key genes in biosensor strains

Deletions of *ISU1*, *HOG1* and *IRA2* were recently shown to epistatically improve anaerobic xylose metabolism in XI-containing strains of *S. cerevisiae* and the effect was reproducible in two different *S. cerevisiae* strain backgrounds (BY4741 and CEN.PK113-5D) [40]. In the current study, xylose utilizing strains from our previously established XR/XDH biosensor panel (TMB375X; [39]) were used to evaluate the effect of *isu1*Δ, *hog1*Δ, and *ira2*Δ on the three main sugar signalling pathways: strains TMB3752 (*HXT1*p-GFP) for the Snf3p/Rgt2p pathway, TMB3755 (*SUC2*p-GFP) for SNF1/Mig1p pathway and TMB3757 (*TPS1*p-GFP) for the cAMP/PKA pathway, Fig. 1a. The strain TMB3751 (same background but without biosensor) was used as a control strain. All biosensor strains were derived from *S. cerevisiae* W303-1A, which is a common host for signalling studies in *S. cerevisiae* [46–48]; CEN.PK strains, on the other hand, are less suitable for sugar sensing studies since they are known to have accumulated mutations in the cAMP/PKA network [49, 50], whereas this is not the case in W303 [51].

In total, 24 strains were constructed for the study (Table 1). The deletions were performed as single and combinatorial knock-outs. Single gene deletions (*isu1*Δ, *hog1*Δ and *ira2*Δ) were made in each of the three previously mentioned biosensor strain lines (TMB3752 (*HXT1*p-GFP), TMB3755 (*SUC2*p-GFP), and TMB3757 (*TPS1*p-GFP)) as well as the control strain (TMB3751; no biosensor). Strains TMB376X (*ira2*Δ) and TMB377X (*isu1*Δ) were used as the background to construct the double deletion (*ira2*Δ*isu1*Δ and *isu1*Δ*hog1*Δ) strains, respectively, and TMB379X strains (*ira2*Δ*isu1*Δ) were used to generate the triple deletion strains (*ira2*Δ*isu1*Δ*hog1*Δ), see Table 1. All deletions were confirmed by yeast colony PCR (data not shown). The effect of the deletions were then analysed in terms of strain characterization and fermentation profiles, and the biosensor signals were measured by flow cytometry and used to assess how the deletions affected the *S. cerevisiae* sugar signalling network.

**Table 1:** *S. cerevisiae* biosensor strains constructed and/or utilized in this study

Strains	Biosensor	Relevant genotype	References
TMB375X series (background strains)			
TMB3751	Control	can1::YIp211; *SPB1/PBN1::YIp128GAL2mut*; *Vac17/MRC1::TKL*-*TAL*; *Chr X*-*2/XI*-*5/XII*-*4::XR*-*XDH*-*XK*	[39]
TMB3752	*HXT1*p	can1::YIpGFP-Hxt1p; *SPB1/PBN1::YIp128GAL2mut*; *Vac17/MRC1::TKL*-*TAL*; *Chr X*-*2/XI*-*5/XII*-*4::XR*-*XDH*-*XK*	
TMB3755	*SUC2*p	can1::YIpGFP-Suc2p; *SPB1/PBN1::YIp128GAL2mut*; *Vac17/MRC1::TKL*-*TAL*; *Chr X*-*2/XI*-*5/XII*-*4::XR*-*XDH*-*XK*	
TMB3757	*TPS1*p	can1::YIpGFP-Tps1p; *SPB1/PBN1::YIp128GAL2mut*; *Vac17/MRC1::TKL*-*TAL*; *Chr X*-*2/XI*-*5/XII*-*4::XR*-*XDH*-*XK*	
TMB361X series			
TMB3761	Control	TMB3751; *ira2*Δ	This study
TMB3762	*HXT1*p	TMB3752; *ira2*Δ	
TMB3765	*SUC2*p	TMB3755; *ira2*Δ	
TMB3767	*TPS1*p	TMB3757; *ira2*Δ	
TMB377X series			
TMB3771	Control	TMB3751; *isu1*Δ	This study
TMB3772	*HXT1*p	TMB3752; *isu1*Δ	
TMB3775	*SUC2*p	TMB3755; *isu1*Δ	
TMB3777	*TPS1*p	TMB3757; *isu1*Δ	
TMB378X series			
TMB3781	Control	TMB3751; *hog1*Δ	This study
TMB3782	*HXT1*p	TMB3752; *hog1*Δ	
TMB3785	*SUC2*p	TMB3755; *hog1*Δ	
TMB3787	*TPS1*p	TMB3757; *hog1*Δ	
TMB379X series			
TMB3791	Control	TMB3761 (*ira2*Δ); *isu1*Δ	This study
TMB3792	*HXT1*p	TMB3762 (*ira2*Δ); *isu1*Δ	
TMB3795	*SUC2*p	TMB3765 (*ira2*Δ); *isu1*Δ	
TMB3797	*TPS1*p	TMB3767 (*ira2*Δ); *isu1*Δ	
TMB385X series			
TMB3851	Control	TMB3771 (*isu1*Δ); *hog1*Δ	This study
TMB3852	*HXT1*p	TMB3772 (*isu1*Δ); *hog1*Δ	
TMB3855	*SUC2*p	TMB3775 (*isu1*Δ); *hog1*Δ	
TMB3857	*TPS1*p	TMB3777 (*isu1*Δ); *hog1*Δ	
TMB386X series			
TMB3861	Control	TMB3791 (*ira2*Δ*isu1*Δ); *hog1*Δ	This study
TMB3862	*HXT1*p	TMB3792 (*ira2*Δ*isu1*Δ); *hog1*Δ	
TMB3865	*SUC2*p	TMB3795 (*ira2*Δ*isu1*Δ); *hog1*Δ	
TMB3867	*TPS1*p	TMB3797 (*ira2*Δ*isu1*Δ); *hog1*Δ	

### The reported improvements of *ira2*Δ, *hog1*Δ and *isu1*Δ with the XI pathway also occurred in strains with the XR/XDH pathway

#### Anaerobic conditions

In the original study of the deletants [40], rich medium (YPX; Yeast extract, Peptone and Xylose) was used for the evaluation. Whereas this is a good strategy to assess the fermentation process performance, it is not necessarily the most suited medium for physiological characterisation. In contrast, a defined medium ensures that the cell will synthesise all its required components in vivo instead of taking it up from a rich medium [52]. Therefore, we performed the anaerobic evaluation of the three combinatorial deletions (*ira2*Δ, *ira2*Δ*isu1*Δ, *ira2*Δ*isu1*Δ*hog1*Δ) in both YPX and YNBX (Yeast Nitrogen Base Xylose; a defined medium) in order to be able to compare to the previous results with the XI strain while making an in-depth physiological characterisation. As a proof-of-concept and to keep the number of strains down to a manageable amount, the effects of the deletions were assessed in the *SUC2*p-GFP biosensor line (TMB37X5; Table 1).

**Fig. 2:**
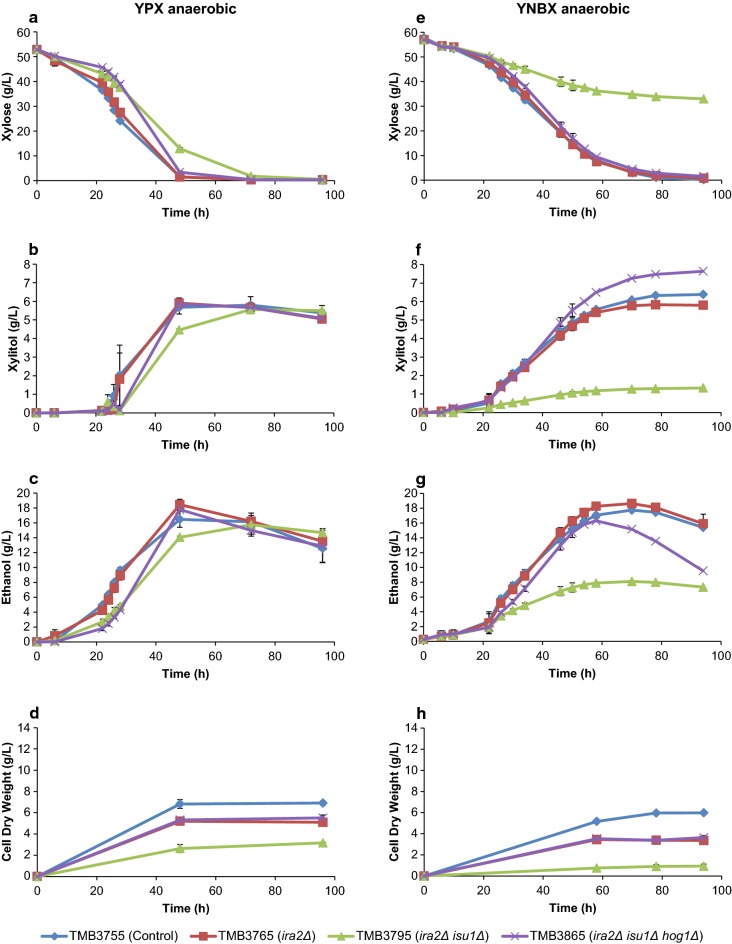
Metabolite and biomass profiles of the anaerobic cultivations of the single, double and triple deletion and control strains. **a**–**d** Anaerobic cultivations in rich medium with xylose (YPX); **e**–**h** anaerobic cultivations in defined medium with xylose (YNBX). Error bars represent the standard deviation between the biological duplicates. The Y-axis scale has been harmonized with Fig. 3 to facilitate comparison, except for Ethanol which was significantly lower in the aerobic conditions (Fig. 3)

In both media and under anaerobic conditions, increased *specific rates* of xylose consumption and ethanol production were obtained in the deletion strains compared to the control strain (Table 2). In parallel, the anaerobic biomass titre decreased with each sequential deletion compared to the control strain (Fig. 2d, h). As for ethanol titres, *ira2*Δ (TMB3765) had the highest maximum titre in YPX and YNBX (Fig. 2c, g), although there was no change in ethanol yield (Table 2). Deletion of *ISU1* in the *ira2*∆ background negatively impacted the xylose, xylitol, ethanol and biomass titres, whereas the additional deletion of *HOG1* (*ira2*Δ*isu1*Δ*hog1*Δ) was partly able to recover the decrease in titre caused by *ira2*Δ*isu1*Δ (Fig. 2). For comparison, Sato et al. observed a different trend for growth since they reported an increased growth rate with each sequential deletion; however, biomass and ethanol titres were not reported [40].

**Table 2:** Anaerobic specific rates and yields in YPX and YNBX media

Strain	Specific xylose consumption rate g/(g CDW L h)	Specific xylitol formation rate g/(g CDW L h)	Specific ethanol formation rate g/(g CDW L h)	Yield ethanol per xylose g/g	Yield xylitol per xylose g/g
YPX (0–48 h)					
TMB3755 (background strain)	1.57 ± 0.09	0.17 ± 0.00	0.51 ± 0.09	0.32 ± 0.04	0.11 ± 0.01
TMB3765 (*ira2*∆)	2.06 ± 0.05	0.24 ± 0.02	0.74 ± 0.04	0.36 ± 0.01	0.12 ± 0.01
TMB3795 (*ira2*∆ *isu1*∆)	3.22 ± 0.56	0.36 ± 0.07	1.14 ± 0.22	0.35 ± 0.01	0.11 ± 0.00
TMB3865 (*ira2*∆ *isu1*∆ *hog1*∆)	1.94 ± 0.09	0.23 ± 0.01	0.70 ± 0.02	0.36 ± 0.01	0.12 ± 0.00
YNBX (0–70 h)					
TMB3755 (background strain)	1.41 ± 0.14	0.16 ± 0.02	0.45 ± 0.01	0.32 ± 0.03	0.11 ± 0.00
TMB3765 (*ira2*∆)	2.19 ± 0.09	0.24 ± 0.01	0.75 ± 0.07	0.34 ± 0.02	0.11 ± 0.00
TMB3795 (*ira2*∆ *isu1*∆)	4.23 ± 0.93	0.24 ± 0.05	1.50 ± 0.18	0.36 ± 0.04	0.06 ± 0.00
TMB3865 (*ira2*∆ *isu1*∆ *hog1*∆)	2.12 ± 0.01	0.29 ± 0.00	0.60 ± 0.05	0.28 ± 0.03	0.14 ± 0.00

The cultivations were performed in two biological replicates. Additional cultivation rates can be found in Additional file 1: Tables S1, S2

A stepwise increase in specific xylose consumption rate was previously observed with each additional deletion in the XI strain [40]. In the present study, the consumption rates of the deletion strains all increased compared to the control, but peaked in the *ira2*Δ*isu1*Δ strain, and not in the triple deletion strain (Table 2; both in YPX and YNBX). In fact, the specific xylose consumption and ethanol formation rates in the *ira2*∆*isu1*∆ strain on YNBX were roughly three times higher than that of the background strain (Additional file 1: Table S2), as a consequence of the severe decrease in biomass formation (Fig. 2d, h), since the specific consumption rate is normalized to the biomass concentration. This implies that the double deletion strain shunted more carbon away from biomass formation and towards the product, as is seen in the specific ethanol productivity of this strain compared to the others (Table 2). In terms of yield of ethanol from xylose, there was no significant change in TMB3795 (*ira2*Δ*isu1*Δ) compared to the control. From a process point-of-view, this strain actually had the worst combination of deletions, since it decreased the *volumetric* consumption and productivity (Additional file 1: Tables S1, S2).

When cultivated on YNBX, the double deletion strain TMB3795 (*ira2*Δ*isu1*Δ) showed a significantly lower fitness and overall performance compared to the other three strains (Fig. 2e–h). This strain clearly benefited from the rich nature of YPX (Fig. 2a–d), and struggled to cope with the minimal medium. It is also evident that fermentation on YNBX (Fig. 2e, f) took longer time than on YPX (Fig. 2a–d)—70 h vs. 48 h to peak Ethanol, for instance; Fig. 2—although the trends were similar. Due to the undefined nature of some of the rich medium components, it was decided to pursue the study with YNBX only.

#### Aerobic conditions

**Fig. 3:**
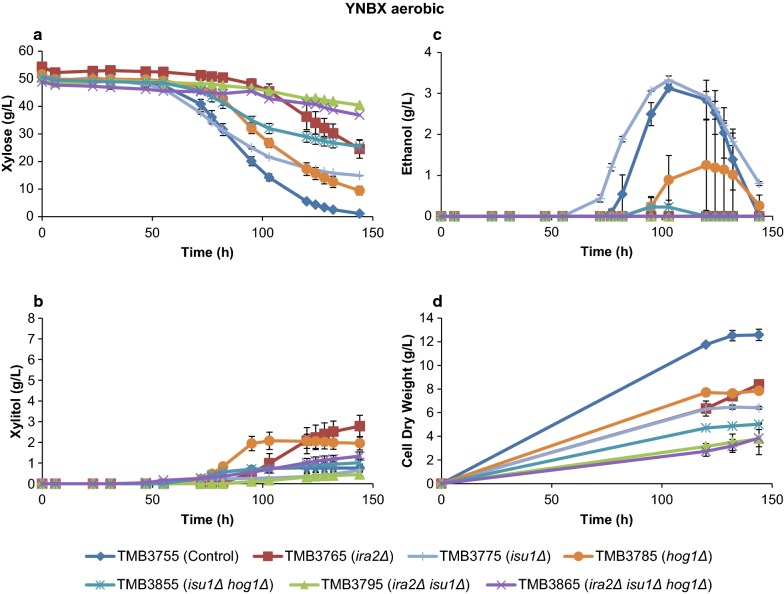
Metabolite and biomass profiles of the aerobic cultivations of the deletion and control strains. **a**–**d** Anaerobic cultivations in defined medium with xylose (YNBX). Error bars represent the standard deviation between the biological duplicates. The Y-axis scale has been harmonized with Fig. 2 to facilitate comparison, except for Ethanol which was significantly lower in the aerobic conditions (this figure)

It was previously suggested that during aerobic conditions, the *isu1*Δ single deletion relieved the starvation response on xylose and that *isu1*Δ*hog1*Δ further improved the specific xylose consumption rate [40]. To be able to investigate whether this also applied to the XR/XDH strains, three more strains were assayed in addition to the four that were used anaerobically: TMB3775 (*isu1*Δ), TMB3785 (*hog1*Δ), TMB3855 (*isu1*Δ*hog1*Δ). The aerobic results on YNBX with the seven strains are shown in Fig. 3. As expected from respiratory growth, the aerobic cultivations in general led to higher biomass yields, but every deletion led to a decrease in final cell dry weight compared to the control strain. The lag time was significantly longer than during anaerobiosis, which has been observed before in XR/XDH strains engineered with a XR with preference for NADH [53].

*isu1*Δ was the best deletion in XR/XDH strains in terms of improved aerobic specific xylose consumption rate (Additional file 1: Table S3). However, any combination that included *isu1*Δ (*isu1*Δ*hog1*Δ, *ira2*Δ*isu1*Δ, *ira2*Δ*isu1*Δ*hog1*Δ) turned out to be worse than the single deletion in terms of specific rates of xylose and ethanol (Additional file 1: Table S3), thereby infirming any epistatic effect of *isu1*Δ*hog1*Δ in the XR/XDH background. The results also demonstrated that the *ira2*Δ single deletion severely decreased the aerobic fitness in every regard: poor xylose consumption, increased xylitol accumulation and decreased production of ethanol and biomass (Fig. 3), which corroborates previous results [40]. It is also notable that, except for TMB3775 (*isu1*Δ) and TMB3785 (*hog1*Δ), no ethanol was detected in the aerobic cultures of the deletion strains (Fig. 3; Additional file 1: Table S3), which indicates that this is either a secondary effect of the decreased rate of xylose consumption (Fig. 3), or that these deletions makes the yeast Crabtree-negative during growth on xylose.

### Signalome responses to *ira2*Δ, *hog1*Δ and *isu1*Δ

**Fig. 4:**
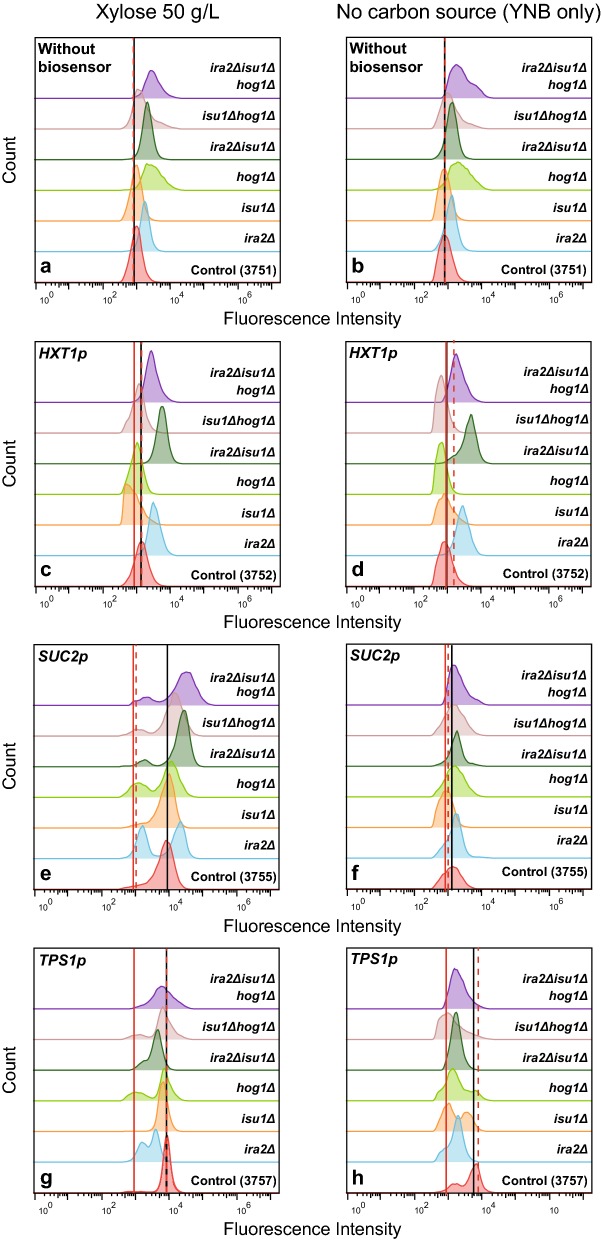
Flow cytometry results on xylose 50 g/L (**a**, **c**, **e** and **g**) and no carbon source—YNB only (**b**, **d**, **f** and **h**) after 6 h.  The histograms represent: strains without biosensor (**a** and **b**), *HXT1*p (**c** and **d**), *SUC2*p (**e** and **f**) and *TPS1*p (**g** and **h**), respectively. Each control strain (**a** and **b**: 3751, **c** and **d**: 3752, **e** and **f**: 3755 and **g** and **h**: 3757) is presented with all its deletion derivatives: single (*isu1*Δ, *hog1*Δ, and *ira2*Δ), double (*ira2*Δ*isu1*Δ and *isu1*Δ*hog1*Δ) and triple (*ira2*Δ*isu1*Δ*hog1*Δ) deletion. The black line indicates the autofluorescence of each control strain. The red dotted line shows the Fluorescence Intensity (FI) of the repression condition of each control strain (see Additional file 1: Figures S2–S4 (0 h)). The red solid line indicates the autofluorescence of control strain 3751 under the same condition (**a**, **b**). The cultivations were performed in oxygen limited microtiter plates, but the results are highly similar to those of the anaerobic and aerobic shake flasks (Additional file 1: Figure S6 )

**Fig. 5:**
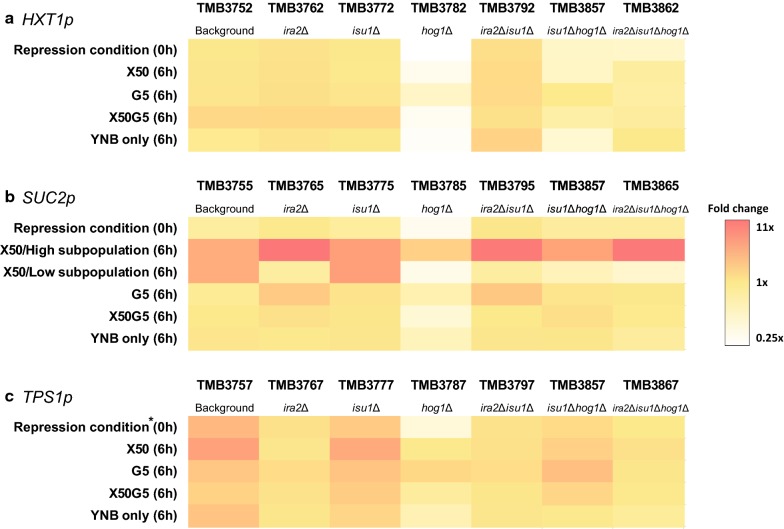
Heat map of the biosensor Fluorescence Intensity (FI) normalized to the autofluorescence of each background strain (TMB37X1; no biosensor). **a** HXT1p, **b** SUC2p and **c** TPS1p. Each biosensor was normalized to the corresponding deletion in the TMB37X1 strains to account for changes in autofluorescence due to changes in morphology caused by the deletions, e.g. TMB3772 (*HXT1p)* was normalized to TMB3771 (no biosensor). The conditions were: repression according to [39], xylose 50 g/L (X50), glucose 5 g/L (G5) and YNB without carbon source (YNB only). *SUC2p* (**b**) displayed subpopulations during cultivation on xylose 50 g/L in some of the strains, and therefore one row for each subpopulation is displayed for X50; note that TMB3755 and TMB3775 only had one population, which is indicated by the same colour in both subpopulation rows. This figure should be interpreted along Fig. 4, since the heat map does not take the strength and potential constitutive expression of the FI signal into account, only the fold change. *The *TPS1*p-GFP biosensor has previously been shown to be difficult to repress in the background strain [38, 39], and does therefore have a fold change > 1 in the 0 h repression condition in TMB3757

Following up on the proposed epistatic interactions between *ira2*Δ, *isu1*Δ and *hog1*Δ [40], the effect of these deletions on the three main sugar signalling pathways were investigated. The TMB3751 strain that lacks any GFP-coupled biosensor was used as a control in order to determine the background fluorescence intensity of the biosensor strains (autofluorescence) and how this was affected by potential changes in cell size and morphology caused by the mutations. When analysing TMB3751 and its derivatives either in the presence of xylose 50 g/L or without any carbon source (YNB only), an autofluorescence increase was observed for the strains with *HOG1* and *IRA2* single deletions, in *hog1*Δ and *ira2*Δ in combination with *isu1*Δ, as well as in the triple deletion case (Fig. 4a). It was also observed that the tendency to flocculate increased with each subsequent deletion, and that the average cell morphology also changed from yeast-shaped to a circular shape (Additional file 1: Figure S5 ), which has previously been reported for e.g. loss-of-function mutants of *IRA2* [54]. Therefore, it was also of importance to normalize each signal to the autofluorescence of each background strain (TMB37X1; no biosensor) to account for effects linked to changes in morphology. The fluorescence results are illustrated in the form of a heat map with the fold change from the corresponding background strain (with the same deletion) to each strain and condition (Fig. 5); e.g. TMB3772 (*HXT1p)* was normalized to TMB3771 (no biosensor) and so on. We also acknowledged that the change in morphology/flocculation tendency made OD measurement unreliable, since it led to increases in apparent OD; instead, we used cell dry weight to quantify biomass, which circumvented the morphology issue. The signalling profiles were very similar in the aerobic, anaerobic conditions and microtiter plates experiments (Additional file 1: Figure S6), and for the sake of throughput, the results of Figs. 4 and 5 are from the microtiter plates (micro-aerobic conditions) only.

### *ira2*∆*isu1*∆ simultaneously leads to high and low glucose signals in different sugar signalling pathways

As was shown in the characterisation section above, *ira2*∆*isu1*∆ resulted in the highest anaerobic specific xylose consumption rate. On the biosensor level, it was observed that *ira2*∆*isu1*∆ constitutively induced the *HXT1*p biosensor (usually activated by high glucose concentrations) in all the conditions, including low glucose (5 g/L), high xylose (50 g/L) and the repressing conditions (Fig. 5a and Additional file 1: Figure S2). The *high glucose*-signal from *ira2*∆*isu1*∆ displayed by the *HXT1*p biosensor (Snf3p/Rgt2p signalling pathway) was also confirmed with the *TPS1*p biosensor (cAMP/PKA pathway) that is normally inducible in low glucose and repressed in high glucose [38] (Additional file 1: Figure S4). *TPS1*p-GFP was repressed by *ira2*∆ and *ira2*∆*isu1*∆ not only in the repression condition, but also in conditions that induced the background strain (TMB3757), for instance, low glucose (G5) and high xylose (X50); Fig. 5c. Thus, *ira2*∆*isu1*∆ promoted a high glucose signal even in low glucose conditions or in conditions that previously gave a low glucose signal (i.e. xylose 50 g/L) [39].

Paradoxically, *SUC2*p, a biosensor that is induced by low glucose signals [38], was also highly induced by *ira2*∆*isu1*∆ on xylose. Therefore, on xylose, *ira2*∆*isu1*∆ both conferred a low glucose signal, (as shown by the *SUC2*p biosensor), and a high glucose signal (*HXT*1p induction and *TPS1*p repression), but in different signalling pathways (SNF1/Mig1p, Snf3p/Rgt2p vs. cAMP/PKA). In fact, the *ira2*∆ and *ira2*∆*isu1*∆ biosensors were induced on xylose in the *HXT1p* and *SUC2p* biosensor strains and downregulated in *TPS1p* when compared to their corresponding background strains (TMB375X), Fig. 5. Taken together, the biosensor analysis and the anaerobic fermentation data showed that all three signalling pathways (Snf3p/Rgt2p, SNF1/Mig1p and cAMP/PKA) were simultaneously activated by *ira2*∆*isu1*∆ in the high xylose condition (X50).

The single deletion of *IRA2* exhibited two distinct subpopulations for the *SUC2*p biosensor in xylose 50 g/L, with cell counts evenly distributed among them (left subpopulation: 43.8% of total cell count; right subpopulation: 56.2%; Fig. 4e-*ira2*∆). Nonetheless, the additional deletion of *ISU1* strongly alleviated the left subpopulation compared to the single *ira2*∆. A similar effect was observed among *isu1*∆ and *hog1*∆ and its combination.

### *hog1*Δ weakens the high glucose signal triggered by *ira2*∆*isu1*∆

While *ira2*∆*isu1*∆ constitutively induced the *HXT1*p biosensor (Fig. 4c), the single deletion of *HOG1* resulted in a repression of *HXT1*p-GFP in almost all of the assayed conditions (Fig. 5a and Additional file 1: Figure S2). This repression also occurred in combination with *isu1*∆ (TMB3852: *isu1*∆*hog1*∆) and to some extent with *ira2*∆*isu1*∆ (TMB3862: *ira2*∆*isu1*∆*hog1*∆). In terms of fluorescence intensity (FI) signal, the triple deletion did not give the same high glucose signal as *ira2*∆*isu1*∆ (Fig. 4). At the same time, the addition of *hog1*∆ in TMB3792 (*ira2*∆*isu1*∆) did not counteract the *SUC2*p *low glucose*-signal conferred by *ira2*∆*isu1*∆ since the major population of *SUC2*p biosensor on TMB3865 (*ira2*∆*isu1*∆*hog1*∆) was still highly induced in all conditions, including xylose 50 g/L (Fig. 4e and Additional file 1: Figure S3). This high FI from the *SUC2*p biosensor implies that the triple deletion strain maintained the same *low glucose*-signal as the *ira2*∆*isu1*∆ strain (*and* the background strain TMB3755, cf. [39]), independently of carbon source. Finally, the impact of *hog1*∆ on *TPS1p* biosensor was mainly observed on the mixture of xylose 50 g/L and glucose 5 g/L, which seemed to keep this biosensor in an induced state (Fig. 5c; Additional file 1: Figure S4).

## Discussion

In the current study, we showed that the epistatic interactions identified between *IRA2*, *ISU1* and *HOG1* gene deletions in a XI strain [40] were also valid for XR/XDH engineered *S. cerevisiae* strains. However, the biggest increase in specific xylose consumption and -ethanol formation rates occurred in the *ira2*∆*isu1*∆ strains (and not in the *ira2*∆*isu1*∆*hog1*∆ strains as in [40]). Also the increase in specific rates was mostly connected to a corresponding decrease in final biomass, and not to an increase in volumetric rates. The present study also showed that there was a clear medium effect when using YPX (rich medium) that overshadowed some of the physiological effects of the deletions, and that defined medium (e.g. YNBX) should be preferred for this type of study. Furthermore, the clear impact of the deletions on the signalome confirmed our previous hypothesis that the efficiency of xylose uptake is connected to one or several of the sugar signalling routes [39].

### Is there a connection between the increase in anaerobic specific xylose consumption rate and the simultaneous signalling of high and low glucose?

**Fig. 6:**
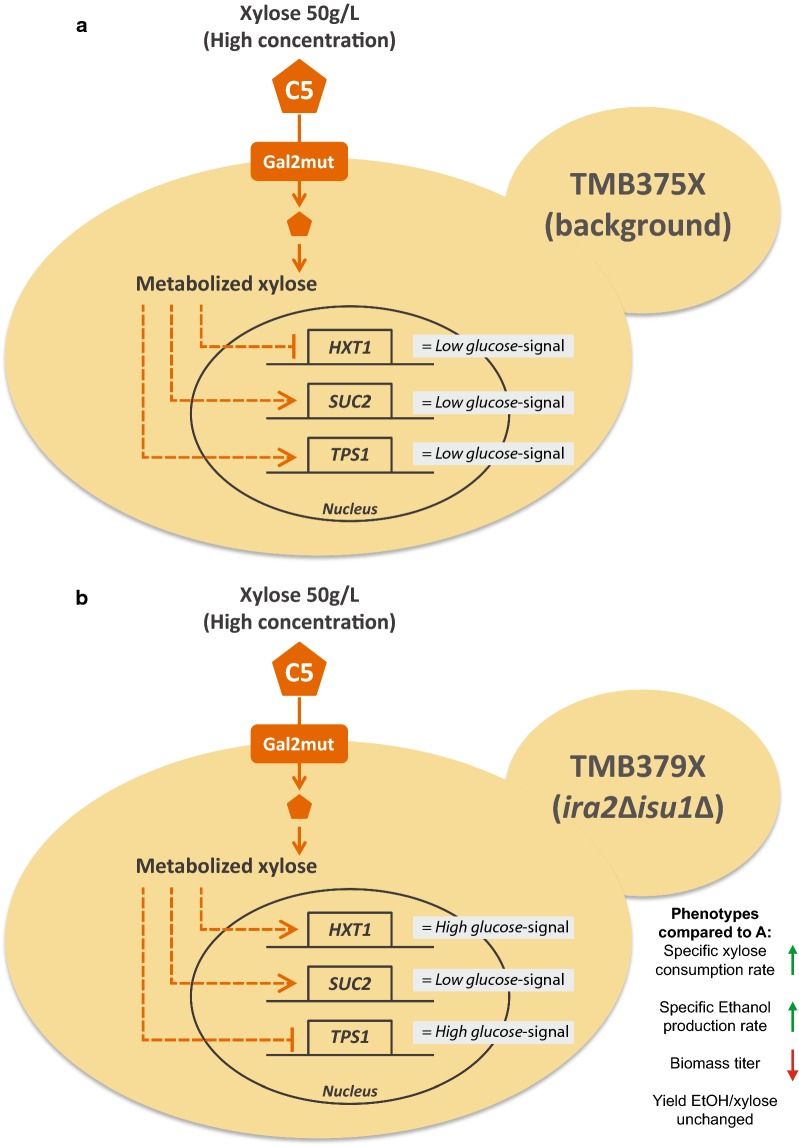
Schematic representation of the signalling effects of xylose on **a** the background strain line, and **b** on the *ira2*∆*isu1*∆ double deletion. High xylose concentrations resulted in a *low glucose*-signal in all the biosensors in the background strain (**a**), but this was not the case for *ira2*∆*isu1*∆ (**b**). Solid arrows: reactions/transport; dashed arrows: induction (arrowhead) or repression (hammerhead). **a** was adapted from our previous work [39]. *HXT1* belongs to the Snf3p/Rgt2p pathway, *SUC2 to* the SNF1/Mig1p pathway and *TPS1* to the cAMP/PKA pathway (Fig. 1)

Previously, we have shown that high xylose concentrations gives the same response as low glucose concentrations in the three sugar signalling pathways in *S. cerevisiae* engineered with the XR/XDH pathway [39]. This *low glucose*-signal on xylose was hypothesised to be one of the reasons why xylose is not recognised as a fermentable carbon source by XR/XDH engineered *S. cerevisiae* [39]; presented schematically in Fig. 6a. In light of this hypothesis, the results with the *ira2*∆*isu1*∆ strains are noteworthy since the deletions do not only improve the specific xylose and ethanol rates, but also change the signalling to confer a *high glucose*-signal in the Snf3p/Rgt2p and cAMP/PKA pathways (*HXT1*p and *TPS1*p biosensors, respectively), while maintaining the *low glucose*-signal in the SNF1/Mig1p pathway (*SUC2*p biosensor), see Fig. 6b. The improved specific rates in the *ira2*∆*isu1*∆ strains are directly related to the lower biomass production and the question that remains is whether the decreased biomass is a consequence of the changes in the signalome. This led us towards the cAMP/PKA pathway, which is known to regulate cell cycle progression, proliferation and homeostasis [55], and is a common denominator for the *ira2*∆ and *hog1*∆ deletions (Fig. 1b).

In very general terms, the level of active PKA in *S. cerevisiae* is glucose-regulated, and is high during growth on glucose and low during growth on alternative carbon sources [55]. cAMP and PKA levels are controlled intracellularly through the RAS-complex and extracellularly through glucose sensing by Gpr1p [46, 47]. Ira1/2p are regulators of RAS that induce the change of RAS from its active, cAMP-promoting form (RAS-GTP) to its inactive form (RAS-GDP) [41, 56]. *ira2*∆ has been reported to lead to constitutively activated RAS-GTP, which in turn leads to elevated levels of cAMP and PKA [46, 57], see Fig. 1b. Mechanistically, *ira2*∆ diminishes the self-regulatory feedback loop of PKA [46], which allows for continuous production of cAMP. This implies that *ira2*∆-containing strains should have elevated cAMP/PKA levels compared to control strains, which fits with the overall *high glucose*-signal in the *TPS1p* biosensor strains (as indicated by *TPS1p* repression; Fig. 5c). PKA activation is dependent on cAMP, which is formed from ATP [58, 59]. In a study on the effect of benzoic acid on *S. cerevisiae*, it was found that addition of moderate levels of benzoic acid in the medium led to increased glycolytic and respiratory rates to compensate for the ATP consumed by pumping protons out of the cell, and to lower biomass [60]. In our case, the observed decrease in biomass production and increased sugar uptake rate in strains with *ira2*∆ in single and combinatorial deletion (Fig. 2h) could therefore be related to a constant ATP drainage from constitutive cAMP/PKA signals that should be very costly in terms of ATP.

Boosted PKA levels have been correlated with a number of phenotypes, some of which were also seen in the deletion strain of the current study. To name a few: PKA leads to lower levels of stress signals through its repression of the *MSN2/4* stress response genes [61], increased pseudohyphal growth [62] and increased critical cell size needed for budding [63] (Fig. 1b). The two latter in particular have also been reported for *ira2*∆ strains along with increased flocculation [54], and could explain the overall change in morphology observed in the *ira2*∆-containing strains (Additional file 1: Figure S5). It is possible that the changes in cell morphology and increased flocculation in the deletion strains are more pronounced in the W303 strain background that was used in the present study. W303 has, in comparison to the gold standard *S. cerevisiae* S288C genome, a number of non-synonymous mutations in flocculation genes that may make it more prone to flocculation [51]. PKA also contributes to the induction of low-affinity hexose transporters (i.e. *HXT1*) by phosphorylation of the Rgt1p protein, thus counteracting the repressive effect of Rgt1p on the low-affinity hexose transporter genes [39, 64]. This is not the only signal needed to induce *HXT1* [48, 65], but it is a supporting mechanism towards the *high glucose*-signal on xylose seen in the *ira2*∆*isu1*∆ strains (Figs. 4 and 6b).

While the above cAMP/PKA reasoning can explain the signal and phenotype of *ira2*∆, it is clear that *isu1*∆ in conjunction with *ira2*∆ reinforces the *high*- and *low glucose* signal. *ISU1* encodes a mitochondrial Fe-S cluster protein and its deletion leads to accumulation of mitochondrial iron, which has been suggested to play a role in cellular iron homeostasis [66]. Deletion of *ISU1* is known to decrease the activity of respiratory enzymes that require Fe–S clusters, and thus results in poor respiratory growth [44], which is also evident from Fig. 3d. It has been reported that *isu1*∆ helped maintain low levels of reactive oxygen species (ROS) during ethanol stress (which was not the case of other Fe–S cluster deletants) [67] and that the elevated mitochondrial iron helped suppressing oxidative damage in *S. cerevisiae* cells lacking the *SOD1* superoxide dismutase [66]. This is likely to be a contributing factor to the enabled xylose respiration that was shown in *isu1*∆ strains when the oxidative phosphorylation was experimentally blocked [40].

The anaerobic effect of *isu1*∆—or rather its effect together with *ira2*∆ (Fig. 2)—are more puzzling, as the mitochondrion is primarily involved in respiration. Also, proteomics analysis of the *isu1*∆ XI strains revealed a difference compared to the control strain during aerobic conditions, but not during anaerobic conditions [40]. The mitochondrial Isu proteins have however been suggested to also be necessary for maturation of cytosolic Fe–S clusters [68]. Since *isu1*∆ changes the iron homeostasis by accumulation of mitochondrial iron [66], it is likely that these cells have a constitutive iron stress. High iron levels inactivate a transcription factor (Aft1p) by migrating it from the mitochondria to the cytosol, meaning that it cannot induce its targets, which include *ISU1/2* and metal transporters [69], and would thus contribute to a decrease in Fe–S cluster formation. How this would lead to the observed decrease in biomass formation and increased specific ethanol production of the current study is difficult to say, but it could be speculated that the cell would benefit from producing ethanol during the iron stress, so that it can grow on it when the stress is relieved. This would however require more experiments than the current study.

It is also noteworthy that *SUC2* is still induced in *ira2*∆*isu1*∆ strains, indicating that there is a *low glucose* signal to this pathway—this was in fact, observed in all of the deletion strains (Figs. 4 and 5). Whereas the *high glucose* signal to the other two signalling pathways suggest that the deletions do tune the cell towards understanding that xylose is fermentable (Fig. 6b), the *low glucose* signal to the SNF1/Mig1p pathway as indicated by the *SUC2*p induction, suggests that yeast could simultaneously sense xylose as a non-fermentable carbon source. Aerobically, the comparative proteomics results of Sato et al. suggested that the *isu1*∆ XI strains did not emit a starvation response on xylose which is normally seen in *S. cerevisiae*, and that the mechanisms for non-fermentable carbon sources were repressed [40]. However, our *SUC2*p biosensor strains suggest that there indeed was an induction signal for the alternative carbon pathways during aerobic conditions (Fig. 4e). *SUC2*p transcription has been found to be induced by PKA during low glucose levels, and repressed by PKA during absence of glucose [70], i.e. PKA can both induce and repress *SUC2p*. The likely elevated PKA levels in the *ira2*∆*isu1*∆ strains in the current study, coupled with proposed *low glucose*-signal from xylose in the XR/XDH background [39] could therefore explain why the *SUC2*p biosensors are induced in the *ira2*∆*isu1*∆ double deletants.

The biggest difference between the current study and that of Sato et al. is that the *ira2*∆*isu1*∆*hog1*∆ triple deletion was not the best strain in terms of specific xylose and ethanol rates but *ira2*∆*isu1*∆ was. The main signalling difference between these two deletions was that *HXT1*p was more induced in the *ira2*∆*isu1*∆ than in *ira2*∆*isu1*∆*hog1*∆ strain (Fig. 4), which could imply that it is of essence to have high expression of low affinity hexose transporters (e.g. *HXT1*p) in order to improve the specific xylose consumption. It should however be noted that the triple deletion did recover the decreases in concentration and volumetric rates of the double deletion (Fig. 4; Additional file 1: Table S2), meaning that the *ira2*∆*isu1*∆*hog1*∆ strains might still be of interest from a process point-of-view.

### Are *ira2*∆ and *isu1*∆ desired genotypes for anaerobic xylose fermentation processes?

In the previous section we hypothesize that the simultaneous *high*- and -*low glucose* signals were one of the reasons for the improved xylose phenotypes of the *ira2*∆*isu1*∆ strains. However, this comes with clear physiological drawbacks:  they consume less xylose, produce less ethanol and biomass (Fig. 4) and tend to flocculate (Additional file 1: Figure S5). The *ira2*∆ single deletion results in an improved strain in terms of specific rates because of the decrease in biomass production. Anaerobic cultivations lead to significantly lower biomass production than aerobic cultivations and *ira2*∆ decreases an already low biomass concentration to even lower levels. This explains why the anaerobic xylose, xylitol and ethanol titres are roughly the same between the control and *ira2*∆ strains while the specific rates increased. As *ira2*∆ leads to constitutive activation of PKA, the cell will have a repressed stress response system (e.g. *MSN2/4*) and thus feel less stress; however, this is a “false happiness”, since the strain cannot really activate its stress system. Lignocellulose hydrolysate is rich in inhibitory compounds such as furans and aromatics, and *ira2*∆ might therefore be a less suitable deletion for this type of fermentation process. The XR/XDH pathway does have an edge over XI here, as XR has 5-hydroxymethyl-furfural detoxifying effect [71]. Adding the *isu1*∆ deletion, that according to literature leads to hyperaccumulation of mitochondrial iron [66], will likely trigger an iron stress and Fe–S cluster insufficiencies, that possibly is masked by the repression of the stress response system by the high PKA levels. Dos Santos et al. who also discovered the effects of the *isu1*∆ the same year as Sato et al. hypothesized that *isu1*∆ might be beneficial to XI strains since XI is a metalloenzyme [45], which is also the case for yeast XDHs [72]. Therefore *ira2*∆*isu1*∆ is a double edged sword: aerobically it is the worst combination in terms of fitness and titres (Fig. 3), but anaerobically it is one of the best in terms of specific rates (Table 2).

The discussed deletions are undeniably a step in the right direction for improving xylose utilization and signalling in *S. cerevisiae.* But the physiological drawbacks warrant us to ask what a desired genotype for xylose fermentation would look like. It is likely that a balance is needed for signalling that may be difficult to achieve by null mutants alone. Turning specific genes on or off is likely to kill or damage the cell, and a more careful gene attenuation would probably be beneficial, e.g. by CRISPRi methods [73]. The biosensor system for monitoring the sugar signalling pathways will be a valuable tool towards this end, as it allows modifications to be assessed by both physiological characterization and signalling patterns.

## Conclusions

We previously showed that xylose is not sensed extracellularly by *S. cerevisiae* [38] and that XR/XDH engineered strains gave a *low glucose*-signal when grown in high xylose concentrations, implying low PKA activity [39]. In the present study, the deletions of *IRA2* and *ISU1* were used to look further into this hypothesis. We were able to show that the deletions did convey xylose fermentation improvements in XR/XDH strains, and that this phenotype was linked to changes in the sugar signalome. The simultaneous *high*- and *low glucose* signal achieved by *ira2*∆*isu1*∆ on xylose 50 g/L suggests that these deletions to some extent alleviate the *low glucose*-signal we saw in the parental XR/XDH biosensor strains [39], and that genetic modifications of this kind are likely a step towards making *S. cerevisiae* recognize xylose as a fermentable carbon source.

## Materials and methods

### Strains and media

The *S. cerevisiae* strains used in the present study are based on previously constructed strains that contain biosensors that couple the promoters from different genes regulated by the sugar signalling pathways to a green fluorescent protein (GFP) [38, 39]. In the current study, the TMB375X series of strains (Table 1; [39]) were used to further study the genetics of the signalome. Besides the different GFP biosensors, the TMB375X strains contain a single-copy of the mutated *GAL2* transporter from the pRS62N_GAL2_N376F plasmid [24], overexpression of two pentose phosphate pathway genes (*TAL1*, *TKL1*) and three copies of XR/XDH/XK xylose pathway [39]. All the strains used in the study are listed in Table 1.

The yeast strains were maintained on Yeast Peptone Dextrose (YPD; 10 g/L yeast extract, 20 g/L peptone, 20 g/L glucose). Physiological characterization was performed in Yeast Peptone (YP) and/or Yeast Nitrogen Base (YNB; 6.7 g/L Yeast Nitrogen Base without amino acids [Becton–Dickinson and Company, USA] buffered with 50 mM potassium hydrogen phthalate at pH 5.5) media supplemented with different concentrations of glucose and/or xylose as a carbon source (see details below). For sub-cloning of plasmids, *Escherichia coli* NEB5-α (New England BioLabs, Ipswich, MA, US) was used and was cultivated in Lysogeny Broth (LB) medium (10 g/L tryptone, 5 g/L yeast extract, 5 g/L NaCl, pH 7.0). Yeast mineral medium (3 g/L KH_2_PO_4_, 0.5 g/L MgSO_4_·7H_2_O, 6.6 g/L K_2_SO_4_, 1 mL/L trace elements, 1 mL/L vitamin solution, pH 6.0 adjusted with KOH) [60] was used for the amdSYM transformations [74]. When using solid plates, 15 g/L of agar was added to the medium. All strains were stored in 25% (v/v) glycerol at − 80 °C and, when relevant, transformed cells were also stored with their corresponding antibiotics.

### Molecular biology methods

Standard methods were used to perform cloning experiments [75]. The primers used in the study, acquired from Eurofins MWG Operon (Ebersberg, Germany), are listed in Additional file 1: Table S4. Competent *E. coli* cells were prepared and transformed according to the methods of Inoue and colleagues [76]; transformants were selected for in LB medium supplemented with 50 μg/mL of ampicillin. The Lithium Acetate transformation protocol [77] with the addition of DMSO (10% v/v) prior to heat shock [78] was used for the *S. cerevisiae* transformations. All yeast transformants were selected according to their specific selection marker and verified by colony PCR [79].

### Deletion of *ISU1* and *HOG1* with CRISPR–Cas9

#### Construction of gRNA plasmids

The pCfB3496 (hphMX marker) [80] and LWA26 (natMX marker) [39] plasmids were used as templates to construct gRNA plasmids to target *ISU1* and *HOG1.* The plasmids were PCR amplified using Phusion High-Fidelity DNA Polymerase (Thermo Fisher Scientific, Waltham, MA, USA) and phosphorylated primers. The forward primers (105_ISU1_f and 105_HOG1_f) contained a 5′ tail with the new 20 bp targeting sequence (Additional file 1: Table S4**)**, and were used together with the 103_r reverse primer to generate the plasmids to target *ISU1* and *HOG1* respectively. The amplicons were purified with GeneJET PCR purification kit (Thermo Fisher Scientific) according to the manufacturer’s protocol. The purified products were digested with *Dpn*I followed by plasmid ligation with T4 DNA ligase (Thermo Fisher Scientific). The obtained plasmids were propagated in *E. coli*, extracted with the GeneJET Plasmid Miniprep Kit (Thermo Fisher Scientific) and verified by Sanger sequencing (Eurofins MWG Operon) using standard primer T3. The final plasmids were named gRNA_ISU1 and gRNA_HOG1 (Additional file 1: Table S5). Two different selection markers were chosen for these plasmids to avoid marker recycling after yeast transformation: gRNA_ISU1 had Hygromycin B (hphMX) and gRNA_HOG1 had ClonNAT (natMX).

#### Construction of donor DNA and deletion of *ISU1* and *HOG1*

A fragment of the bacterial AmpR ampicillin resistance gene (330 bp) was amplified to be used as a junk-donor DNA to delete *ISU1* and *HOG1* with CRISPR–Cas9. Primers with 50 bp tails with homology to the upstream and downstream regions of *ISU1* (primers Amp_ISU1_f and Amp_ISU1_r) and *HOG1* (primers Amp_HOG1_f and Amp_HOG1_r) were used to amplify the fragment. Agarose gel electrophoresis was performed to confirm the presence of the desired PCR products. The fragment was purified with the GeneJET PCR purification kit (Thermo Fisher Scientific).

All yeast strains used in this study already had the pCfB2312 plasmid containing a Cas9 gene [80]. The strains were transformed by the addition of 1 µg gRNA plasmid and 0.5 µg of donor DNA, specific for each deletion. *ISU1* and *HOG1* transformants were selected on YPD plates with 200 μg/mL geneticin supplemented with either 200 μg/mL Hygromycin B or 100 μg/mL clonNAT, respectively.

### Deletion of *IRA2* with amdSYM

*IRA2* proved difficult to delete with the CRISPR–Cas9 system, which was possibly due to the large size of this gene (9240 bp). Instead, the amdSYM strategy [74] was used for this deletion. The amdSYM cassette was amplified from the pUG-amdSYM plasmid [74] with IRA2_S1_amdSYM_f and IRA2_S2_amdSYM_r primers containing 50 bp homology to the upstream and downstream region of *IRA2*. The fragment was purified with the GeneJet PCR purification kit (Thermo Fisher Scientific) and 1 µg purified product was used for the yeast transformations. The transformants were selected for growth on mineral medium [60] with 0.6 g/L acetamide; the same medium but with 2.3 g/L fluoroacetamide was used to recycle the amdSYM marker. Recycling of amdSYM marker was accomplished by inoculating a single colony in 10 mL of YPD in a 50 mL of conical centrifuge tubes at 30 °C, 180 rpm followed by plating on mineral medium [60] with 2.3 g/L fluoroacetamide. Colony PCR was used to verify the recycling.

### Flow cytometry

Single-cell fluorescence intensity (FI) was measured with a BD Accuri C6 flow cytometer in connection with a BD CSampler autosampler (Becton–Dickinson, NJ, US). Detection was done at 488 nm and 533/30 bandpass filter (FL1-H channel). Pre-cultivations, induction/repression conditions and sample preparations were performed as previously described [38, 39]. Cells were inoculated in a microtiter plate starting with an OD_620nm_ = 0.5 and were incubated for 6 h, at 800 rpm and 30 °C. Different media were tested during the incubation: YNB-KHPthalate medium with glucose 1 g/L, 5 g/L and 40 g/L; xylose 50 g/L; xylose 50 g/L combined with glucose 5 g/L and with no carbon source (YNB only). The threshold was set to 800 at FL1-H channel and 10 000 events were collected per sample. Flow cytometry data from technical and biological replicates were analyzed with the FlowJo v10 software (Treestar, Inc., San Carlos, CA).

### Aerobic and anaerobic cultivations

Prior to the aerobic and anaerobic fermentation, pre-cultivations were carried out as in the flow cytometry experiment [39]. The aerobic cultivations were performed with 100 mL of YNB-KHPthalate supplemented with 50 g/L of xylose (YNBX) in 1 L baffled shake flasks. For the anaerobic cultivations, YNB-KHPthalate supplemented with 50 g/L of xylose (YNBX) and YP supplemented with 50 g/L of xylose (YPX) were used, respectively. 100 mL media were added to 1 L non-baffled shake flasks sealed with a curved neck attached to a rubber stopper. Nitrogen gas was sparged through an inlet port connected to a 0.22 μm sterile filter. Glycerol (2 mL) was added into the curved neck to maintain the anaerobic environment while allowing for gas release. A separate outlet was used for sampling.

All cultivations (anaerobic and aerobic) were performed at 30 °C and 180 rpm and in biological duplicates. Samples for optical density at 620 nm (OD_620_), metabolites, and flow cytometry (at 0 h and 6 h) were taken and stored at 4 °C. The samples were not frozen since it has been observed that storage at − 20 °C negatively affects the quantification of high xylose concentrations.

### Biomass and metabolite analysis

Biomass was determined both as OD_620_ with an Ultrospec 2100 Pro spectrophotometer (Amersham Biosciences, Uppsala, Sweden) and as cell dry weight (CDW). CDW was performed by vacuum filtering 5 mL culture through pre-weighted Supor 450 Membrane Disc Filters (0.45 μm; Pall Corporation, NY, USA), followed by washing with distilled water and drying for 8 min at 350 W in a microwave. The dried filters were stored in a desiccator prior to weighing.

A Waters HPLC system (Milford, MA, USA) was used to quantify extracellular metabolites in the cultivations. The system was run with an Aminex HPX-87H ion exchange column (Bio-Rad, Hercules, CA, USA) at 60 °C, and a mobile phase of 5 mM H_2_SO_4_ flowing at 0.6 mL/min. Compounds were detected with a refractive index detector (Waters model 2414; Milford, MA, USA).

## Additional file

**Additional file 1.** Additional Methods, Figures and Tables.

## Data Availability

Flow cytometry and fermentation data are available upon request.

## Abbreviations

CDW: cell dry weight
FI: fluorescence intensity
GFP: green fluorescent protein
OD: optical density
PCR: polymerase chain reaction
XDH: xylitol dehydrogenase
XI: xylose isomerase
XK: xylulokinase
XR: xylose reductase
YNB: Yeast Nitrogen Base
YNBX: Yeast Nitrogen Base Xylose
YPD: Yeast extract Peptone Dextrose
YPX: Yeast extract Peptone Xylose
