# Supplementary material for: Exploring the xylose paradox in Saccharomyces cerevisiae through in vivo sugar signalomics of targeted deletants
Source: Microb Cell Fact. 2019 May 23;18:88. doi: 10.1186/s12934-019-1141-x (PMC6532234; doi:10.1186/s12934-019-1141-x)
Supplement: Supplementary file 1 — Additional file 1. Additional Methods, Figures and Tables. [file 12934_2019_1141_MOESM1_ESM.docx]

Additional Information for:

Exploring the xylose paradox in *Saccharomyces cerevisiae* through *in vivo* sugar signalomics of targeted deletants

Karen O Osiro, Celina Borgström, Daniel P Brink, Birta Líf Fjölnisdóttir and Marie F Gorwa-Grauslund*

Applied Microbiology, Department of Chemistry, Lund University, Lund, Sweden

*Corresponding author

E- mail: [marie-francoise.gorwa@tmb.lth.se](mailto:marie-francoise.gorwa@tmb.lth.se)

# Additional Methods

**Uncompensated and compensated carbon balances**

The carbon balances for the anaerobic and aerobic shake flask cultivations were calculated based on the measured concentrations of xylose, xylitol, glycerol, acetate and ethanol in C-mols/L (Table S1-3), see Eq. 1. C_n_ in the equation is the number of C-mols of compounds *n,* and one C‐mol biomass of *S. cerevisiae* was approximated with CH_1.62_O_0.47_N_0.21_ [[1](#_ENREF_1)]. A degree-of-reduction (DR) balance was made in parallel to solve the equation system (Eq. 2), with each coefficient being the degree-of-reduction of the compound.

$$C_{xylose}= C_{xylitol}+C_{glycerol}+C_{acetate}+C_{EtOH}+C_{biomass} (Eq. 1)$$

$$4{\cdot C}_{xylose}= {4.4\cdot C}_{xylitol}+{4.67\cdot C}_{glycerol}+4\cdot C_{acetate}+6\cdot C_{EtOH}+4.11\cdot C_{biomass} (Eq. 2)$$

Since the anaerobic DR balances only closed around 90% (Table S1-3), a compensated balance was made where it was assumed that there were two missing compounds: Ethanol and CO_2_ and that their addition would close the DR balance to 100%. The missing degree-of-reduction (∆DR) and missing carbon balance (∆C) in the measured data was thus as assumed to consist of:

$$\Delta DR={DR}_{in}-{DR}_{out}=6\cdot C_{EtOH}+0\cdot C_{{CO}_{2}} (Eq. 3)$$

$$\Delta C=C_{in}-C_{out}=C_{EtOH}+C_{{CO}_{2}} (Eq. 4)$$

where 6 and 0 are the degree-of-reduction coefficients of Ethanol and CO_2_ respectively. The compensated balances (Table S1-S3) did improve the Carbon balance closure, but they still only close at 70-75%. This implies that the HPLC quantification did not capture all the carbon, and that there was carbon that probably was lost due to the N_2_-sparging.

# Additional Figures

**Figure S1. Histogram of the control strain (without biosensor) and all its derivatives (TMB 3XX1) at different carbon sources:** pre-culture on G40 (0h), glucose 40 g/L (G40), mixture of xylose 50 g/L and glucose 5 g/L (X50G5) and glucose 5 g/L (G5). Black line indicates the autofluorescence of each control strain. Red dotted line shows the Fluorescence Intensity (FI) of the repression condition of control strain. The experiments were performed in microtiterplates.

**
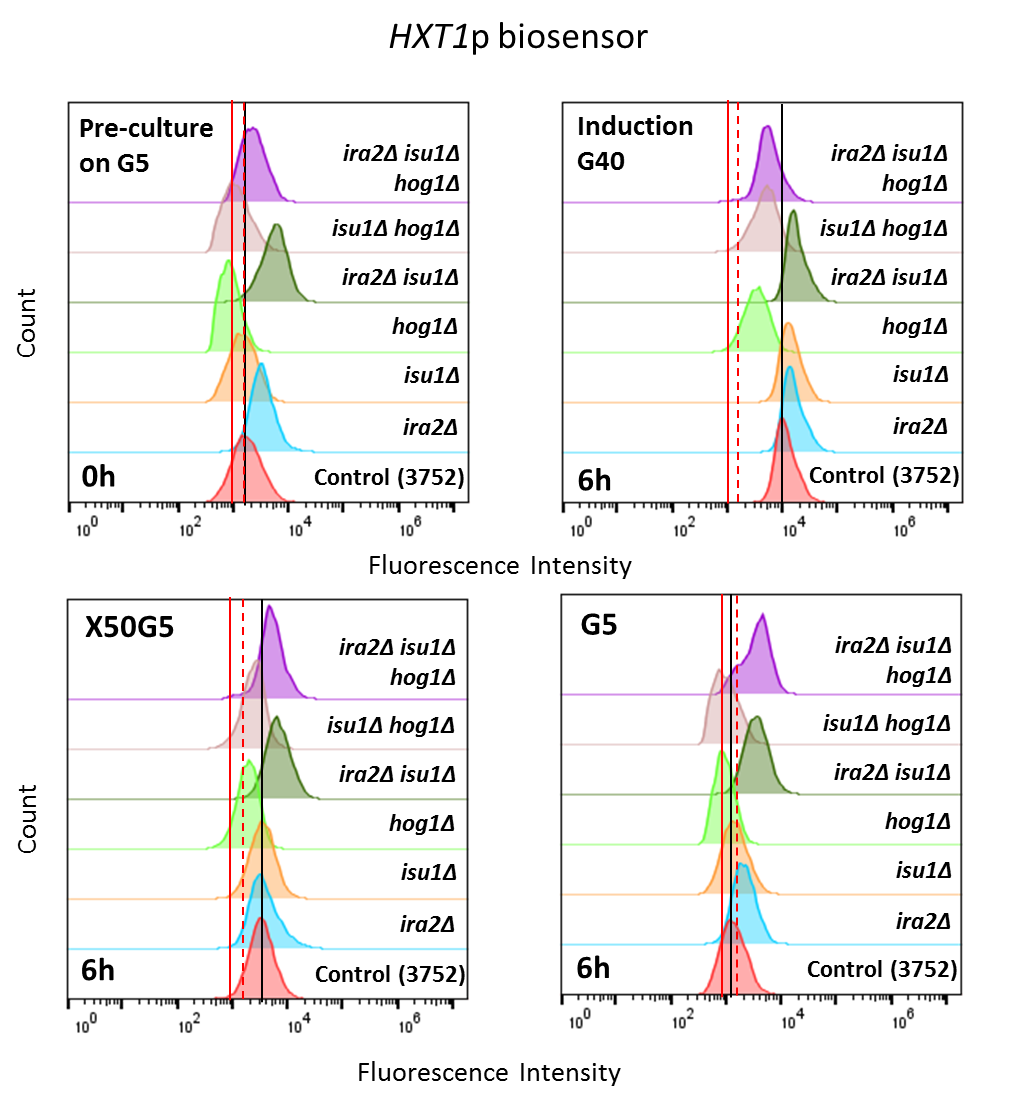
**

**Figure S2. Histogram of the *HXT1*p biosensor and all its derivatives (TMB 3XX2) at different carbon sources:** pre-culture on G40 (0h), glucose 40 g/L (G40), mixture of xylose 50 g/L and glucose 5 g/L (X50G5) and glucose 5 g/L (G5). Black line indicates the autofluorescence of each control strain. Red dotted line shows the Fluorescence Intensity (FI) of the repression condition of control strain. Red solid line indicates the autofluorescence of control strain 3751 under same condition as presented on each graph. The experiments were performed in microtiterplates.

**Figure S3. Histogram of the *SUC2*p biosensor and all its derivatives (TMB 3XX5) at different carbon sources**: pre-culture on G40 (0h), glucose 40 g/L (G40), mixture of xylose 50 g/L and glucose 5 g/L (X50G5) and glucose 5 g/L (G5). Black line indicates the autofluorescence of each control strain. Red dotted line shows the Fluorescence Intensity (FI) of the repression condition of control strain. Red solid line indicates the autofluorescence of control strain 3751 under same condition as presented on each graph. The experiments were performed in microtiterplates.

**Figure S4. Histogram of the *TPS1*p biosensor and all its derivatives (TMB 3XX7) at different carbon sources**: pre-culture on G40 (0h), glucose 40 g/L (G40), mixture of xylose 50 g/L and glucose 5 g/L (X50G5) and glucose 5 g/L (G5). Black line indicates the autofluorescence of each control strain. Red dotted line shows the Fluorescence Intensity (FI) of the repression condition of control strain. Red solid line indicates the autofluorescence of control strain 3751 under same condition as presented on each graph. The experiments were performed in microtiterplates.

**
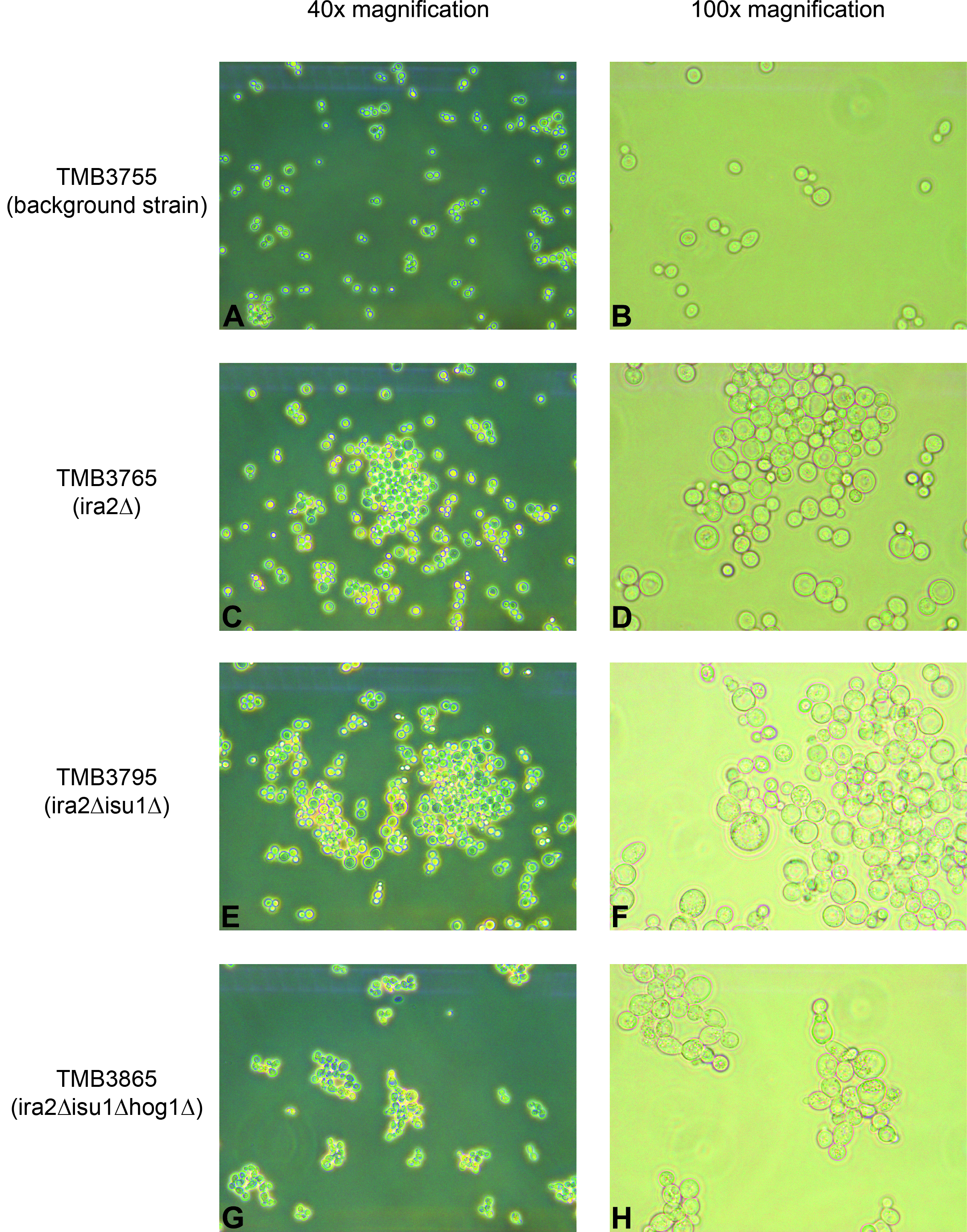
**

**Figure S5. Microscopy photos of the overnight cultures in glucose 40 g/L of the background strain for the *SUC2p* biosensor (TMB3755) and the subsequent deletions of *ira2***∆, ***isu1***∆**, *hog1***∆**.** Note the changes in flocculation tendency and cell morphology with each deletion. Left hand side: 40x magnification; right hand side: 100x magnification. Photos were taken with a Leica ICC50W microscope camera (Leica Microsystems, Germany).

**
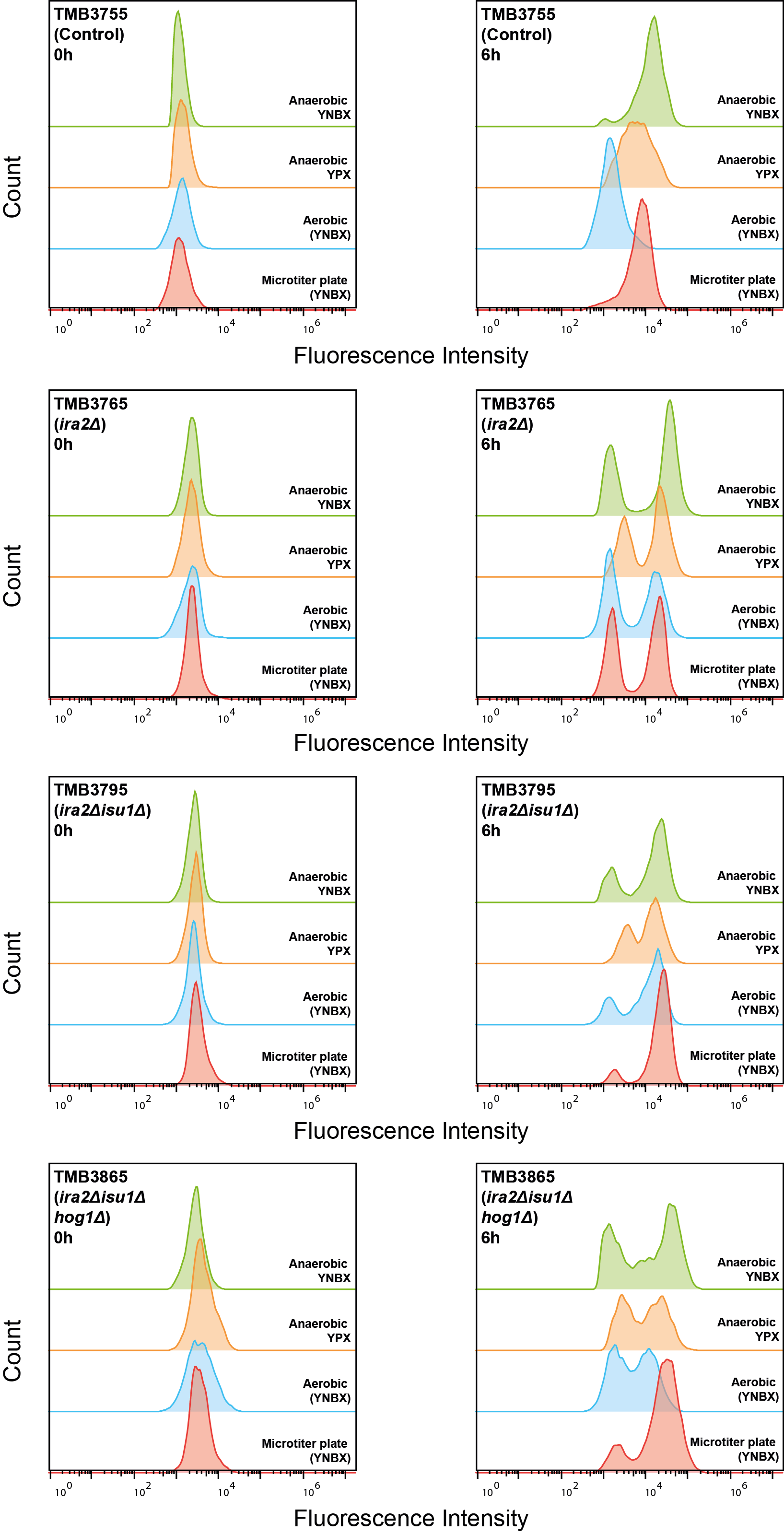
**

**Figure S6. Comparison of the signal of the *SUC2p* biosensors across the anaerobic and aerobic shake flask experiments, and the microtiter plate experiment.** Representative histograms were used for each condition. Note that the signal intensity (X-axis) is comparable across the different oxygenations and vessel volumes, except in the aerobic experiment for the background strain.

# Additional Tables

**Table S1.** Rates, yields and carbon balance data from the **anaerobic** shake flasks with **YPX** (0-48h), performed in two biological replicates.

|  | TMB3755  (background strain) | TMB3765  (∆ira2) | TMB3795  (∆ira2 ∆isu1) | TMB3865  (∆ira2 ∆isu1 ∆hog1) |
| --- | --- | --- | --- | --- |
| Volumetric Xylose consumption rate (g ∙L^-1^ ∙h^-1^) | 10.684 ±0.318 | 10.701 ±0.253 | 8.323 ±0.317 | 10.305 ±0.310 |
| Volumetric Xylitol formation rate (g ∙L^-1^ ∙h^-1^) | 1.182 ±0.107 | 1.230 ±0.081 | 0.930 ±0.015 | 1.218 ±0.012 |
| Volumetric Glycerol formation rate (g ∙L^-1^ ∙h^-1^) | 0.141 ±0.079 | 0.027 ±0.038 | 0.181 ±0.026 | 0.022 ±0.031 |
| Volumetric Acetate formation rate (g ∙L^-1^ ∙h^-1^) | 0.149 ±0.028 | 0.157 ±0.020 | 0.134 ±0.028 | 0.143 ±0.034 |
| Volumetric Ethanol formation rate (g ∙L^-1^ ∙h^-1^) | 3.430 ±0.312 | 3.841 ±0.214 | 2.928 ±0.045 | 3.706 ±0.038 |
|  |  |  |  |  |
| Specific Xylose consumption rate (g ∙g CDW^-1^ ∙L^-1^ ∙h^-1^) | 1.571 ±0.090 | 2.058 ±0.049 | 3.223 ±0.555 | 1.938 ±0.090 |
| Specific Xylitol formation rate (g ∙g CDW^-1^ ∙L^-1^ ∙h^-1^) | 0.173 ±0.001 | 0.237 ±0.016 | 0.361 ±0.070 | 0.229 ±0.006 |
| Specific Glycerol formation rate (g ∙g CDW^-1^ ∙L^-1^ ∙h^-1^) | 0.021 ±0.013 | 0.005 ±0.007 | 0.069 ±0.005 | 0.004 ±0.006 |
| Specific Acetate formation rate (g ∙g CDW^-1^ ∙L^-1^ ∙h^-1^) | 0.022 ±0.006 | 0.030 ±0.004 | 0.053 ±0.022 | 0.027 ±0.006 |
| Specific Ethanol formation rate (g ∙g CDW^-1^ ∙L^-1^ ∙h^-1^) | 0.507 ±0.090 | 0.739 ±0.041 | 1.137 ±0.221 | 0.697 ±0.018 |
|  |  |  |  |  |
| Yield Ethanol per Xylose (g/g) | 0.322 ±0.039 | 0.359 ±0.012 | 0.352 ±0.008 | 0.360 ±0.007 |
| Yield Xylitol per Xylose (g/g) | 0.111 ±0.007 | 0.115 ±0.005 | 0.112 ±0.002 | 0.118 ±0.002 |
| Yield Acetate per Xylose (g/g) | 0.014 ±0.003 | 0.015 ±0.002 | 0.016 ±0.004 | 0.014 ±0.004 |
| Yield Glycerol per Xylose (g/g) | 0.013 ±0.008 | 0.003 ±0.004 | 0.022 ±0.002 | 0.002 ±0.003 |
|  |  |  |  |  |
| Carbon balance closure | 70.52% | 71.29% | 68.07% | 72.30% |
| Degree of reduction closure | 93.14% | 96.15% | 92.64% | 97.25% |
|  |  |  |  |  |
| Compensated Carbon balance closure | 75.09% | 73.86% | 72.97% | 74.13% |
| Compensated Degree of reduction closure | 100.00% | 100.00% | 100.00% | 100.00% |

**Table S2.** Rates, yields and carbon balance data from the **anaerobic** shake flasks with **YNBX** (0-70h), performed in two biological replicates.

|  | TMB3755  (background strain) | TMB3765  (∆ira2) | TMB3795  (∆ira2 ∆isu1) | TMB3865  (∆ira2 ∆isu1 ∆hog1) |
| --- | --- | --- | --- | --- |
| Volumetric Xylose consumption rate (g ∙L^-1^ ∙h^-1^) | 7.810 ±0.319 | 7.657 ±0.069 | 3.162 ±0.635 | 7.489 ±0.322 |
| Volumetric Xylitol formation rate (g ∙L^-1^ ∙h^-1^) | 0.869 ±0.054 | 0.825 ±0.024 | 0.181 ±0.032 | 1.037 ±0.053 |
| Volumetric Glycerol formation rate (g ∙L^-1^ ∙h^-1^) | 0.076 ±0.011 | 0.051 ±0.015 | 0.079 ±0.025 | 0.025 ±0.001 |
| Volumetric Acetate formation rate (g ∙L^-1^ ∙h^-1^) | 0.096 ±0.026 | 0.060 ±0.029 | 0.066 ±0.011 | 0.081 ±0.029 |
| Volumetric Ethanol formation rate (g ∙L^-1^ ∙h^-1^) | 2.496 ±0.100 | 2.623 ±0.105 | 1.118 ±0.115 | 2.128 ±0.288 |
|  |  |  |  |  |
| Specific Xylose consumption rate (g ∙g CDW^-1^ ∙L^-1^ ∙h^-1^) | 1.414 ±0.144 | 2.190 ±0.086 | 4.225 ±0.926 | 2.116 ±0.010 |
| Specific Xylitol formation rate (g ∙g CDW^-1^ ∙L^-1^ ∙h^-1^) | 0.158 ±0.019 | 0.236 ±0.005 | 0.242 ±0.047 | 0.293 ±0.001 |
| Specific Glycerol formation rate (g ∙g CDW^-1^ ∙L^-1^ ∙h^-1^) | 0.014 ±0.003 | 0.014 ±0.004 | 0.105 ±0.035 | 0.007 ±0.001 |
| Specific Acetate formation rate (g ∙g CDW^-1^ ∙L^-1^ ∙h^-1^) | 0.008 ±0.002 | 0.019 ±0.004 | 0.022 ±0.031 | 0.017 ±0.004 |
| Specific Ethanol formation rate (g ∙g CDW^-1^ ∙L^-1^ ∙h^-1^) | 0.451 ±0.010 | 0.751 ±0.066 | 1.492 ±0.181 | 0.600 ±0.053 |
|  |  |  |  |  |
| Yield Ethanol per Xylose (g/g) | 0.320 ±0.026 | 0.343 ±0.017 | 0.357 ±0.035 | 0.284 ±0.026 |
| Yield Xylitol per Xylose (g/g) | 0.111 ±0.002 | 0.108 ±0.002 | 0.058 ±0.002 | 0.138 ±0.001 |
| Yield Acetate per Xylose (g/g) | 0.012 ±0.003 | 0.008 ±0.004 | 0.022 ±0.008 | 0.011 ±0.004 |
| Yield Glycerol per Xylose (g/g) | 0.010 ±0.001 | 0.007 ±0.002 | 0.025 ±0.003 | 0.003 ±0.000 |
|  |  |  |  |  |
| Carbon balance closure | 70.41% | 67.05% | 61.48% | 66.63% |
| Degree of reduction closure | 94.10% | 92.13% | 86.53% | 90.24% |
|  |  |  |  |  |
| Compensated Carbon balance closure | 74.35% | 72.30% | 70.47% | 73.14% |
| Compensated Degree of reduction closure | 100.00% | 100.00% | 100.00% | 100.00% |

**Table S3.** Rates, yields and carbon balance data from the **aerobic** shake flasks with **YNBX** (0-120h), performed in two biological replicates.

|  | TMB3755  (Background strain) | TMB3765  (∆ira2) | TMB3775 (∆isu1) | TMB3785 (∆hog1) | TMB3795  (∆ira2 ∆isu1) | TMB3855  (∆isu1 ∆hog1) | TMB3865  (∆ira2 ∆isu1 ∆hog1) |
| --- | --- | --- | --- | --- | --- | --- | --- |
| Volumetric Xylose consumption rate (g ∙L^-1^ ∙h^-1^) | 3.748 ±0.262 | 1.503 ±0.551 | 2.743 ±0.185 | 2.858 ±0.052 | 0.625 ±0.121 | 1.800 ±0.427 | 0.654 ±0.026 |
| Volumetric Xylitol formation rate (g ∙L^-1^ ∙h^-1^) | 0.065 ±0.024 | 0.174 ±0.074 | 0.029 ±0.007 | 0.171 ±0.040 | 0.026 ±0.021 | 0.074 ±0.013 | 0.085 ±0.025 |
| Volumetric Glycerol formation rate (g ∙L^-1^ ∙h^-1^) | 0.006 ±0.007 | 0.000 ±0.000 | 0.003 ±0.004 | 0.001 ±0.002 | 0.000 ±0.000 | 0.000 ±0.000 | 0.000 ±0.000 |
| Volumetric Acetate formation rate (g ∙L^-1^ ∙h^-1^) | 0.012 ±0.016 | 0.015 ±0.021 | 0.202 ±0.004 | 0.132 ±0.043 | 0.060 ±0.085 | 0.250 ±0.017 | 0.022 ±0.031 |
| Volumetric Ethanol formation rate (g ∙L^-1^ ∙h^-1^) | 0.237 ±0.056 | 0.000 ±0.000 | 0.243 ±0.016 | 0.104 ±0.130 | 0.000 ±0.000 | 0.000 ±0.000 | 0.000 ±0.000 |
|  |  |  |  |  |  |  |  |
| Specific Xylose consumption rate (g ∙g CDW^-1^ ∙L^-1^ ∙h^-1^) | 0.319 ±0.028 | 0.233 ±0.054 | 0.435 ±0.038 | 0.372 ±0.028 | 0.202 ±0.061 | 0.382 ±0.089 | 0.244 ±0.042 |
| Specific Xylitol formation rate (g ∙g CDW^-1^ ∙L^-1^ ∙h^-1^) | 0.005 ±0.002 | 0.027 ±0.008 | 0.005 ±0.001 | 0.022 ±0.004 | 0.009 ±0.008 | 0.016 ±0.003 | 0.033 ±0.016 |
| Specific Glycerol formation rate (g ∙g CDW^-1^ ∙L^-1^ ∙h^-1^) | 0.000 ±0.001 | 0.000 ±0.000 | 0.000 ±0.001 | 0.000 ±0.000 | 0.000 ±0.000 | 0.000 ±0.000 | 0.000 ±0.000 |
| Specific Acetate formation rate (g ∙g CDW^-1^ ∙L^-1^ ∙h^-1^) | 0.001 ±0.001 | 0.002 ±0.003 | 0.032 ±0.000 | 0.017 ±0.007 | 0.021 ±0.029 | 0.053 ±0.004 | 0.009 ±0.013 |
| Specific Ethanol formation rate (g ∙g CDW^-1^ ∙L^-1^ ∙h^-1^) | 0.020 ±0.004 | 0.000 ±0.000 | 0.038 ±0.002 | 0.013 ±0.016 | 0.000 ±0.000 | 0.000 ±0.000 | 0.000 ±0.000 |
|  |  |  |  |  |  |  |  |
| Yield Ethanol per Xylose (g/g) | 0.064 ±0.019 | 0.000 ±0.000 | 0.089 ±0.012 | 0.037 ±0.046 | 0.000 ±0.000 | 0.000 ±0.000 | 0.000 ±0.000 |
| Yield Xylitol per Xylose (g/g) | 0.018 ±0.008 | 0.114 ±0.007 | 0.011 ±0.003 | 0.060 ±0.015 | 0.039 ±0.026 | 0.043 ±0.017 | 0.131 ±0.043 |
| Yield Acetate per Xylose (g/g) | 0.003 ±0.004 | 0.008 ±0.011 | 0.074 ±0.007 | 0.046 ±0.014 | 0.085 0.120± | 0.144 ±0.043 | 0.035 ±0.049 |
| Yield Glycerol per Xylose (g/g) | 0.002 ±0.002 | 0.000 ±0.000 | 0.001 ±0.002 | 0.001 ±0.001 | 0.000 ±0.000 | 0.000 ±0.000 | 0.000 ±0.000 |
|  |  |  |  |  |  |  |  |
| Carbon balance closure | 40.07% | 52.34% | 41.88% | 40.88% | 61.595% | 42.90% | 55.69% |
| Degree of reduction closure | 45.20% | 54.57% | 48.36% | 44.55% | 63.320% | 43.99% | 58.05% |
|  |  |  |  |  |  |  |  |
| Compensated Carbon balance closure | 76.60% | 82.62% | 76.31% | 77.85% | 86.048% | 80.24% | 83.65% |
| Compensated Degree of reduction closure | 100.00% | 100.00% | 100.00% | 100.00% | 100.000% | 100.00% | 100.00% |

**Table S4.** List of the primers that were used in this study. Lowercase underlined sequences indicate gRNA sequences.

| Primer | Sequence 5'-3' | Description | Reference |
| --- | --- | --- | --- |
| LW105 + gRNA | gaaacggtgtcgagtaggtaGTTTTAGAGCTAGAAATAGCAAG | gRNA for IRA2 | This study |
| LW 105 + gRNA | attggtacaggggatgacctGTTTTAGAGCTAGAAATAGCAAG | gRNA for ISU2 | This study |
| LW 105 + gRNA | agccgaataaggatgagccaGTTTTAGAGCTAGAAATAGCAAG | gRNA for HOG1 | This study |
| LWA103 | GATCATTTATCTTTCACTGC | Reverse primer for gRNA | This study |
| IRA2_S1_amdSYM_f | GATATCAACTAAACTGTATACATTATCTTTCTTCAGGGAGAAGCA | HR tail 50bp for IRA2 donor DNA | This study |
| IRA2_S2_amdSYM_r | TACAGATAGATATTGATATTTCTTTCATTAGTTTATGTAACACCT | HR tail 50bp for IRA2 donor DNA | This study |
| IRA2_S1_amdSYM_f | TGAAGCTTCGTACGCTGCAG | Primer for IRA2 donor DNA amplification | This study |
| IRA2_S2_amdSYM_r | GCATAGGCCACTAGTGGATCTG | Primer for IRA2 donor DNA amplification | This study |
| IRA2_f | GACCGATTCCAAGTCATCCC | Verification of internal IRA2 | This study |
| IRA2_r | TGACACAAAGAGAAGGGCAC | Verification of internal IRA2 | This study |
| IRA2_Del_Verif_f | TGACTTGGGTTGGGACTTGG | Verification of IRA2 deletion | This study |
| 6_5'amdSYMup_r | CGAGGAGCCGTAATTTTTGC | Verification of IRA2 deletion | This study |
| 5_3'amdSYMdown_f | CCAGATGCGAAGTTAAGTGC | Verification of IRA2 deletion | This study |
| IRA2_Del_Verif_r | ACAGAAACACTTTCAACTAAGACGG | Verification of IRA2 deletion | This study |
| Amp_ISU1_r | TGCATGAGAAGAGGGAGAGAAAGGAAATGCTAGATTAAGGGAAAGAAATA | HR tail 50bp for ISU1 donor DNA | This study |
| Amp_ISU1_f | TGCACGCTCATGGGTATCAATTGGCTAGGTCTAATATTGTTATTGTTTGG | HR tail 50bp for ISU1 donor DNA | This study |
| Amp_ISU1_r | TCAGCGATCTGTCTATTTCG | Primer for ISU1 donor DNA amplification | This study |
| Amp_ISU1_f | GAATGAAGCCATACCAAACG | Primer for ISU1 donor DNA amplification | This study |
| ISU1_Del_Verif_f | ACAACCAATCTACGGGCACA | Verification of ISU1 deletion | This study |
| ISU1_f | CATCAGACCTGTGAATGCCA | Verification of internal ISU1 | This study |
| ISU1_r | CTTGATCGCATCTTCCGCTA | Verification of internal ISU1 | This study |
| Amp_HOG1_r | CGGTTAAAACAGAAAAAAATCATGATCGAAATACGTCCACTTTACTTTGT | HR tail 50bp for HOG1 donor DNA | This study |
| Amp_HOG1_f | CAAATATTATCTATCGTCGAAATTATCATACTATCTTACAATAAGAGTAG | HR tail 50bp for HOG1 donor DNA | This study |
| HOG1_US_f | CAGCGTTAGTGAGGTGAGGG | Verification of HOG1 deletion | This study |
| Amp_Verif_r | CTACAGGCATCGTGGTGTCA | Verification of deletion | This study |
| Amp_Verif_f | ATCTACACGACGGGGAGTCA | Verification of deletion | This study |
| HOG1_DS_r | TGCATTCCCACATCCACGAA | Verification of HOG1 deletion | This study |
| HOG1_f | GCATTTGGGTTGGTTTGCTC | Verification of internal HOG1 | This study |
| HOG1_r | CCATCACTGCCACCAATCTT | Verification of internal HOG1 | This study |
| LW11 | GAACTTCACCACCTTAGAGC | ALD6 amplification (Positive control of DNA) | [[2](#_ENREF_2)] |
| LW12 | TAGCACCTTGGAAGTTAGCC | ALD6 amplification (Positive control of DNA) | [[2](#_ENREF_2)] |
| IRA2_Del_Verif_f | TGACTTGGGTTGGGACTTGG | Verification of amdSYM recycling | This study |
| IRA2_Del_Verif_r | ACAGAAACACTTTCAACTAAGACGG | Verification of amdSYM recycling | This study |
| 5_3'amdSYMdown_f | CCAGATGCGAAGTTAAGTGC | Verification of amdSYM recycling | This study |

**Table S6.** List of the plasmids used in this study.

| Plasmid | Relevant Genotype | Reference |
| --- | --- | --- |
| pLWA26 | *gRNA-VAC17/MRC1 ; natMX* | [[2](#_ENREF_2)] |
| pCfB2312 | *TEF1p-Cas9-CYC1t; kanMX* | [[3](#_ENREF_3)] |
| pCfB3496 | *SNR52p-gRNA; ADE2-SUP4t; hphMX* | Supplied by Vratislav Stovicek (Technical University of Denmark, DTU) |
| pUG6-amdSYM2 | *AmpR ; AnTEF2p-amdSYM-anTEF2t (from Aspergillus nidulans)* | [[4](#_ENREF_4)] |
| gRNA_*HOG1* | *SNR52p*; gRNA-*HOG1-CYC1t*; *natMX* | This Study |
| gRNA_*ISU1* | *SNR52p; gRNA_ISU1-CYC1t; hphMX* | This Study |
| gRNA_IRA2 | *SNR52p*; gRNA-*IRA2-CYC1t*; *natMX* | This Study |

# References

1. Costenoble R, Adler L, Niklasson C, Lidén G: **Engineering of the metabolism of *Saccharomyces cerevisiae* for anaerobic production of mannitol.** *FEMS yeast research* 2003, **3:**17-25.

2. Osiro KO, Brink DP, Borgström C, Wasserstrom L, Carlquist M, Gorwa-Grauslund MF: **Assessing the effect of d-xylose on the sugar signaling pathways of *Saccharomyces cerevisiae* in strains engineered for xylose transport and assimilation.** *FEMS yeast research* 2018.

3. Stovicek V, Borodina I, Forster J: **CRISPR–Cas system enables fast and simple genome editing of industrial *Saccharomyces cerevisiae* strains.** *Metabolic Engineering Communications* 2015, **2:**13-22.

4. Solis-Escalante D, Kuijpers NG, Nadine B, Bolat I, Bosman L, Pronk JT, Daran J-M, Daran-Lapujade P: ***amdSYM*, a new dominant recyclable marker cassette for *Saccharomyces cerevisiae*.** *FEMS yeast research* 2013, **13:**126-139.
